# The Oncogenic Functions of MASTL Kinase

**DOI:** 10.3389/fcell.2018.00162

**Published:** 2018-11-23

**Authors:** Kamila Marzec, Andrew Burgess

**Affiliations:** ^1^ANZAC Research Institute, University of Sydney, Sydney, NSW, Australia; ^2^Faculty of Medicine and Health, Concord Clinical School, University of Sydney, Sydney, NSW, Australia

**Keywords:** MASTL, mitosis, chromosome instability (CIN), oncogene, cancer biology, AKT pathway

## Abstract

MASTL kinase is a master regulator of mitosis, essential for ensuring that mitotic substrate phosphorylation is correctly maintained. It achieves this through the phosphorylation of alpha-endosulfine and subsequent inhibition of the tumor suppressor PP2A-B55 phosphatase. In recent years MASTL has also emerged as a novel oncogenic kinase that is upregulated in a number of cancer types, correlating with chromosome instability and poor patient survival. While the chromosome instability is likely directly linked to MASTL’s control of mitotic phosphorylation, several new studies indicated that MASTL has additional effects outside of mitosis and beyond regulation of PP2A-B55. These include control of normal DNA replication timing, and regulation of AKT/mTOR and Wnt/β-catenin oncogenic kinase signaling. In this review, we will examine the phenotypes and mechanisms for how MASTL, ENSA, and PP2A-B55 deregulation drives tumor progression and metastasis. Finally, we will explore the rationale for the future development of MASTL inhibitors as new cancer therapeutics.

## The Mastl-ENSA-PP2A Axis

In 2010, MASTL (microtubule-associated serine/threonine kinase-like) was identified as the human orthologue of *Drosophila* Greatwall (Gwl) kinase (Burgess et al., 2010), a protein essential for mitosis (Bettencourt-Dias et al., 2004; Yu et al., 2004). Since then, MASTL has established itself as a critical regulator of cell cycle control and maintenance of mitotic integrity (Bettencourt-Dias et al., 2004; Yu et al., 2004; Burgess et al., 2010; Mochida et al., 2010; Álvarez-Fernández et al., 2013; Diril et al., 2016). Its roles in regulating mitosis have been extensively reviewed (Voets and Wolthuis, 2012; Lorca and Castro, 2013; Vigneron et al., 2016), and hence only a brief summary of these functions is described below.

Cellular entry into mitosis relies on the phosphorylation of thousands of proteins (Burgess et al., 2017), a process which is primarily driven by cyclin-dependent kinase 1 (CDK1), a major component of the M-phase-promoting factor (MPF). Prior to mitosis, Cyclin B-CDK1 dependent phosphorylation on substrates is rapidly removed by the PP2A-B55 phosphatase (Mochida et al., 2009). During mitosis, the activity of PP2A-B55 must be suppressed to ensure CDK1 phosphorylation sites on key proteins, such as MPS1 (Diril et al., 2016), remain phosphorylated. This is achieved by MASTL, which indirectly inhibits PP2A-B55 through phosphorylation of alpha-endosulfine (ENSA) and the highly related cAMP-regulated phosphoprotein 19 (Arpp-19) on a single site [S67/62, respectively (Gharbi-Ayachi et al., 2010; Mochida et al., 2010)]. ENSA is in significant excess to PP2A-B55 and acts as an unfair competitive inhibitor, preventing PP2A-B55 from dephosphorylating other substrates (Williams et al., 2014). Interestingly, multisite phosphorylation of ENSA modulates its PP2A-B55 inhibition, with CDK phosphorylation on T28 creating a weak PP2A-B55 inhibitor, while PKA phosphorylation at S106 antagonizes T28 phosphorylation thereby preventing inhibition of PP2A-B55 (Mochida, 2014). To exit mitosis, cells must reactivate PP2A-B55 and sequentially dephosphorylate substrates in a highly ordered manner (Bouchoux and Uhlmann, 2011). This is achieved by a PP1-PP2A relay switch (Grallert et al., 2014), where loss of CDK1 activity is driven by APC^cdc20^ ubiquitination and destruction of cyclin B. Interestingly, MASTL promotes cyclin B recruitment to the APC/C, which likely helps ensure a robust bistable switch at the metaphase-anaphase transition (Voets and Wolthuis, 2015). Loss of CDK1 activity relieves its inhibitory phosphorylation of T320 on PP1, allowing PP1 to auto-dephosphorylate T320 and subsequently partially dephosphorylate and reduce MASTL activity (Heim et al., 2015; Ma et al., 2016; Rogers et al., 2016a). This releases inhibition of PP2A-B55, which in turn begins removing CDK1 phosphorylation events, including those on MASTL (Rogers et al., 2016a) and MPS1 (Diril et al., 2016), thereby creating a positive feedback loop and a bistable mitotic exit switch. PP2A-B55 and FCP1 then complete the dephosphorylation and deactivation of MASTL during mitotic exit (Hégarat et al., 2014; Monica Della et al., 2015). Importantly, disrupting the MASTL-ENSA-PP2A-B55 (MEP) axis results in multiple mitotic errors, including chromosome segregation and cytokinesis defects (Burgess et al., 2010; Voets and Wolthuis, 2010; Cundell et al., 2013; McCloy et al., 2014). This in turn can drive chromosome instability (CIN), a hallmark of cancer (Bakhoum and Swanton, 2014). Consequently, MASTL is now recognized as a master regulator of mitosis (Vigneron et al., 2016), and a critical and essential component of MPF in eukaryotic cells (Hara et al., 2012). However, until recently, the role of MASTL in human diseases, such as cancer, were poorly understood. In this review, we will explore the recent publications on the role of MASTL deregulation in cancer, the mechanisms by which MASTL may directly and indirectly promote tumorigenesis, and its potential as a therapeutic target.

## Deregulation of the Mastl-ENSA-PP2A Axis in Cancer

**FIGURE 1:**
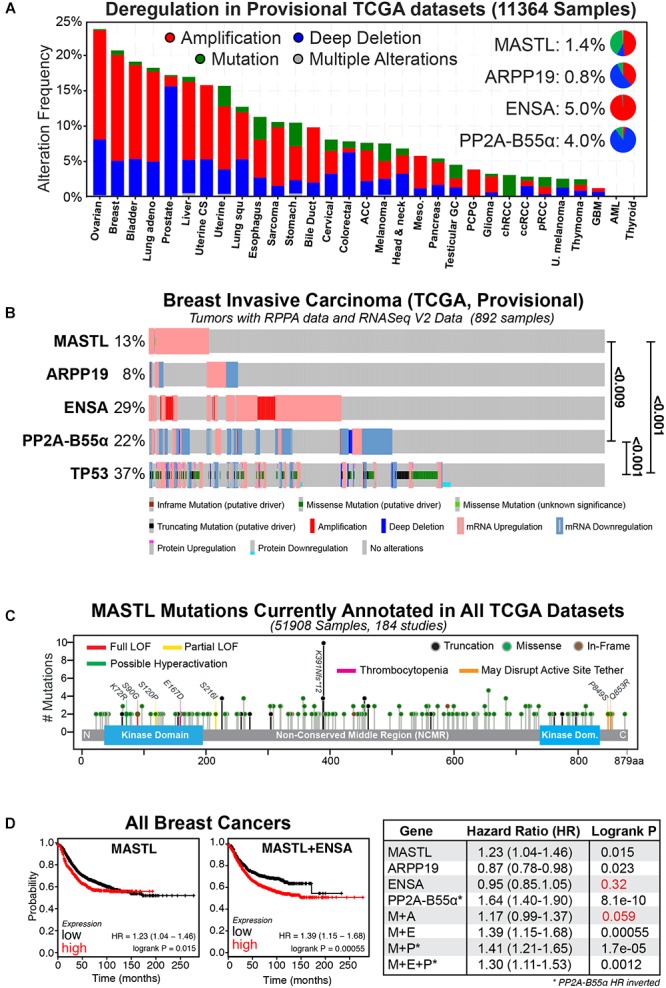
The MASTL-ENSA-PP2A axis in breast cancer. **(A)** The alteration frequencies of MASTL, ENSA, Arpp-19 and PP2A-B55α currently reported in the TCGA provisional tumor datasets for each cancer type are shown and visualized using cBioPortal (http://www.cbioportal.org). Inset pie-charts show the proportion of mutations (green), amplifications (red), and deletions (blue) seen for each gene across all cancers. **(B)** Alterations observed for MASTL, PP2A-B55α (*PPP2R2A*), ENSA, Arpp-19 and p53 (*TP53)* in the current TCGA, Provisional dataset for all complete tumors with RPPA protein data and RNASeq V2 Data (892 samples) in the TCGA provisional Breast Invasive Carcinoma cohort. Significant *p*-values for co-occurrences between gene alterations are shown. **(C)** All known MASTL mutations currently annotated across the entire 184 TCGA studies (51908 samples). Colored lines indicate possible and known functional sites, circled colors indicate mutation type (truncation, missense, or in-frame). **(D)** Kaplan-Meier plots for relapse-free survival were generated using KMPlot (http://kmplot.com) for MASTL, ENSA, Arpp-19, PP2A-B55α (*PPP2R2A*) either alone or in combination for all breast cancer types. Note the hazard ratio (HR) values for PP2A-B55α were inverted in all analyses. Consequently, here low expression of PP2A-B55α correlates with poor survival, while MASTL and ENSA and Arpp-19 HR values correspond to high expression. Non-significant *P*-values are highlighted in red.

PP2A phosphatase is a multimeric complex consisting of a catalytic (C) subunit, a scaffolding (A) subunit, and a regulatory B subunit for which there are 4 members (B, B’, B” and B”’), which each have multiple isoforms. Together, the trimeric (C-A-B) complex can form up to 100 different combinations that regulate a vast number of signaling pathways (Wlodarchak and Xing, 2016). For simplicity, we have limited the remainder of our review to the B55α subunit (herein referred to as B55), as it is a potential tumor suppressor (Ruvolo, 2016) and the primary target of MASTL in human cells. Consequently, overexpression of MASTL could provide an oncogenic growth advantage by functionally repressing the tumor suppressor activity of PP2A-B55. In support, the current provisional TCGA datasets show that the MEP axis is commonly disrupted in a wide-variety of cancer types (Gao et al., 2013). Amplification and deletion are the most commonly observed alterations, while mutations, although present, are rare in most cancer types (Figure 1A). Breast cancer is one of the top cancers that show deregulation of the MEP axis, and this deregulation is further exacerbated from 20 to over 50% by the inclusion of RNA seq and RPPA protein expression data (Figure 1B). There is a clear enrichment for upregulation/amplification of MASTL-ENSA and a corresponding deletion or downregulation of PP2A-B55, matching their proposed oncogenic and tumor suppressor functions. The mutational rate for *MASTL* across all cancers is low and the mutations are evenly spread across the length of the gene (Figure 1C). Most of these mutations have unclear functional consequences, however, there is one annotated breast tumor with a K72R mutation, which may cause hyperactivity similar to the K72M mutant in drosophila (Archambault and Zhao, 2007). There are also several mutations located in or near the N-terminal kinase domain and C-terminal active site tether region, which are known to disrupt activity (Vigneron et al., 2011; Blake-Hodek et al., 2012). There are also several truncating mutations, including a potential truncating hot-spot at K391, which is found in stomach, oesophageal and colon cancers (Figure 1C). A single case of the E167D mutation, which has been linked to thrombocytopenia (Johnson et al., 2009; Hurtado et al., 2018), is annotated in lung squamous cell carcinoma. Understanding what functional impact these mutations and truncations have on MASTL function in cancer will be of interest for future research.

Multiple reports have shown that MEP axis deregulation correlates with various measures of patient outcome in numerous cancer types. Specifically, overexpression of MASTL has been linked with tumor progression and poor outcomes in breast (Álvarez-Fernández et al., 2018; Rogers et al., 2018; Yoon et al., 2018), oral (Wang et al., 2014), gastric (Sun et al., 2017) and colon cancer (Vera et al., 2015; Uppada et al., 2018). The majority of research to date is in breast cancer, where MASTL overexpression correlates significantly with increased chromosome instability, mitotic index, nuclear pleomorphism, histological grade and poor overall survival, (Álvarez-Fernández et al., 2018; Rogers et al., 2018; Yoon et al., 2018), and with a high risk of metastatic relapse in estrogen receptor (ER) positive patients (Zhuge et al., 2017). In contrast, PP2A-B55 is frequently downregulated or deleted in multiple cancer types including AML (Shouse et al., 2016), lung (Kalev et al., 2012), ovarian (Zhang et al., 2018) prostate (Cheng et al., 2011) and breast cancer (Curtis et al., 2012; Beca et al., 2015). In breast cancer, PP2A-B55 inactivation is associated with negative estrogen and progesterone receptor expression, positive HER2 expression, increased proliferative index, higher AKT and ERK phosphorylation, higher grade tumors, faster relapse (Watt et al., 2017), and significantly worse patient outcomes (Figure 1D). Surprisingly, in pancreatic cancer, PP2A-B55 appears to act as an oncogene, with overexpression hyperactivating the AKT, ERK, and Wnt signaling pathways (Hein et al., 2016), indicating that the tumor suppressive role is likely tissue-specific. In contrast, increased expression of ENSA, while common in cancer, has not yet been strongly linked with any cancer patient outcomes. For example, in breast cancer, its overexpression does not affect relapse-free survival (Figure 1D; Gyõrffy et al., 2010). However, when combined with MASTL overexpression, patient outcomes are worse compared to MASTL overexpression alone (Figure 1D). A possible explanation is that increased amounts of both MASTL and ENSA could result in a larger pool of phosphorylated ENSA, inhibiting PP2A-B55 further and creating a functional loss of a tumor suppressor. Notably, the rate of Arpp-19 deregulation is lower, with overexpression providing a small positive advantage that counteracts MASTL (Figure 1D), suggesting it does not play a significant oncogenic role. In summary, MEP axis disruption is a common event in a variety of cancers that correlates with poor patient outcomes. In the following sections we will discuss the specific phenotypes and potential mechanisms for how disruption to the MEP axis drives cancer.

## Effects of Mastl Overexpression

Multiple studies spanning *Drosophila, Xenopus*, and mammalian (mouse and human) systems, have examined MASTL loss of function, clearly establishing its essential role in controlling mitosis (Yu et al., 2004; Burgess et al., 2010; Mochida et al., 2010; Álvarez-Fernández et al., 2013). However, the effects of overexpression are less well understood. Early work in *Drosophila* embryos showed that like loss-of-function, excess MASTL activity could also disrupt mitosis. Work by Archambault et al showed that a K97M hyperactivating mutation (K72M in humans) caused severe developmental defects, characterized by detachment of one centrosome during early prophase (Archambault and Zhao, 2007). Similarly, we recently demonstrated that overexpression of wild-type MASTL in immortalized human MCF10A breast epithelial cells was sufficient to increase the rate of chromosome bridges and micronuclei formation (Rogers et al., 2018). These defects lead to an increase in DNA damage foci and a p38-dependent G2 delay, without disrupting replication dynamics. In addition, MASTL overexpression also disrupted contact inhibition, causing unrestrained growth of MCF10A cells in 3D culture, along with altered migration and a partial epithelial–mesenchymal transition (EMT). In support, Vera et al showed that overexpression in MDA-MB-231 breast cancer cells, which express ∼three-fold more MASTL than MCF-10A cells (Rogers et al., 2018), could drive additional hyperproliferation, invasion and migration in these cells (Vera et al., 2015), suggesting a positive correlation between levels of MASTL and severity of the phenotypic outcomes.

## Effects of ENSA Overexpression

Overexpression of ENSA has been reported to have no proliferative or invasive phenotype in breast cancer cells (Vera et al., 2015) and surprisingly it suppresses tumor growth in liver cells (Chen et al., 2013). These contradictory results combined with the general lack of effect seen in patient data (Figure 1D), suggests that ENSA overexpression is likely to be biologically inert in most cell types and requires activation or inhibition by MASTL or other upstream kinases such as CDK1 and PKA (Mochida, 2014). Despite this, knockdown of ENSA does disrupt normal DNA replication timing (Charrasse et al., 2017), while reduced ENSA expression has been associated with neurodegenerative disease (Ysselstein et al., 2017) and impaired insulin secretion (Bataille et al., 1999). In the case of DNA replication timing, this is dependent on MASTL and PP2A-B55 (Charrasse et al., 2017), highlighting the importance of maintaining an optimal balance between the levels of MASTL-ENSA and PP2A-B55 in mitosis and throughout the cell cycle.

## Effects of PP2A-B55 Loss

In contrast to ENSA, loss of PP2A-B55 has been widely implicated in regulating diverse biological pathways, including neurodegeneration (Taleski and Sontag, 2018), metabolism (Reid et al., 2013), diabetes (Goldsworthy et al., 2016), DNA repair (Kalev et al., 2012; Wang et al., 2015), the cell cycle (Burgess et al., 2017), and of course tumor suppression (Ruvolo, 2016). Importantly, knockdown of PP2A-B55 closely phenocopies MASTL overexpression, with loss of PP2A-B55 in MCF10A cells inducing excessive proliferation resulting in the formation of large lobular acini in 3D culture (Watt et al., 2017). Knockdown of PP2A-B55 also disrupts mitotic exit, with cells delaying during anaphase due to a failure to efficiently dephosphorylate key mitotic substrates, such as PRC1 (Schmitz et al., 2010; Cundell et al., 2016), leading to a disruption to the normally highly ordered dephosphorylation of mitotic substrates (Rogers et al., 2016b). These defects can be mimicked by disrupting the balance between CDK1 and PP2A (McCloy et al., 2014), and importantly the co-knockout of MASTL and PP2A-B55 cancels each other’s mitotic defects (Álvarez-Fernández et al., 2018). However, it should be noted that inhibition of PP2A-B55 has also been reported to suppress mitotic defects and CIN induced by Plk1 overexpression (Cunningham et al., 2016). This could be related to the earlier findings in *Drosophila*, which showed Gwl and Polo (MASTL and Plk1 in humans) play antagonistic mitotic roles (Archambault and Zhao, 2007). Taken together these results highlight the importance of maintaining a tight control over the balance between PP2A-B55 and MASTL to ensure that mitotic phosphorylation and mitotic fidelity is maintained. In support, we showed using mathematical modeling, that MASTL overexpression disrupts PP2A-B55 reactivation timing, delaying mitotic exit (Rogers et al., 2016a; Rogers et al., 2018).

## Regulation of Oncogenic Kinase Signaling by the Mep Axis

MASTL overexpression or loss of PP2A-B55 have been associated with increased proliferation, EMT and invasion in several cancer types including breast, lung and colon cancer. Notably, deregulation of the PI3K/AKT/mTOR pathway, a key regulator of proliferation and EMT, has been directly linked to both MASTL overexpression and PP2A-B55 loss (Kuo et al., 2008), providing a potential mechanistic link. In support, the MEP axis is a well-established regulator of the AKT/mTOR pathway in yeast (Pérez-Hidalgo and Moreno, 2017). In human cells, Vera et al showed that MASTL overexpression led to AKT hyperactivation through increased S473 phosphorylation. However, a recent publication by Aìlvarez-Fernaìndez et al. (2018) was unable to find a correlation between MASTL overexpression and S473 phosphorylation in breast cancer cell lines, while we did observe a small, weak correlation in a larger panel of breast cancer cell lines and tumor samples (Rogers et al., 2018). The answer is likely to be highly dependent on additional genetic defects present in individual cancer cells. The AKT pathway contains multiple positive and negative feedback loops that are often disrupted in cancer (Janku et al., 2018), potentially explaining the differential correlation between MASTL overexpression and AKT activation. For example, PP2A-B55 has been reported as a negative regulator of AKT activity in acute myeloid leukemia (Shouse et al., 2016), and a positivity regulator in pancreatic cancer (Hein et al., 2016).

Adding to the confusion is that it is still unclear exactly how MASTL regulates the AKT pathway. Vera et al proposed an indirect mechanism, independent of PP2A-B55, whereby S473 phosphorylation is increased through GSK3β dependent degradation of the S473 phosphatase PHLPP (Vera et al., 2015). The mechanism for MASTL regulation of GSK3β is currently unknown. To add further confusion, PP2A was recently implicated in dephosphorylation of S9 on GSK3β (Chu et al., 2016), suggesting that this pathway could still be partly dependent on PP2A-B55 inhibition. Interestingly, MASTL was also recently shown to promote Wnt/β-catenin signaling in colon cancer by regulating GSK3β S9 phosphorylation (Uppada et al., 2018). Further highlighting the substantial signaling cross-talk, PP2A-B55 has also been implicated as a negative regulator of β-catenin phosphorylation, with knockdown of PP2A-B55 resulting in increased β-catenin phosphorylation and decreased Wnt signaling (Zhang et al., 2009). We also observed significant disruption to members of the Wnt pathway upon MASTL overexpression, including increased phosphorylation of β-catenin and mislocalization of E-cadherin. It will be important in future research to tease apart the specific signaling pathways and cross-talk to determine if these effects are dependent on MASTL’s inhibition of PP2A-B55, or through the as yet undetermined mechanism for regulation of GSK3β.

An alternative possibility is that MASTL may have additional substrates beyond ENSA and regulation of PP2A-B55. In yeast, Rim15p, the orthologue of human MASTL, is capable of phosphorylating additional substrates, including the nutrient-responsive transcription factors Msn2p/4p and Hsf1p, during starvation (Lee et al., 2013). Here, Rim15 plays an important role in negatively regulating TORC1 (mTOR in humans) signaling under nutrient stress conditions by promoting degradation of G1 cyclins, stabilization of mRNA and promoting a G0 transitional program through phosphorylation of Msn2/4 and Hsf1p (Pérez-Hidalgo and Moreno, 2017). We did observe decreased phosphorylation of mTOR and increased phosphorylation of its downstream targets p70S6 kinase and RPS6 (Rogers et al., 2018), supporting the possibility that MASTL may directly regulate the mTOR pathway in human cells. It will be of great interest to determine if and how well conserved this function is in higher eukaryotes and human cells, and if this can explain how MASTL overexpression results in deregulation of the AKT/mTOR pathway in cancer.

## Working Model of Mastl Driven Cancer Evolution

**FIGURE 2:**
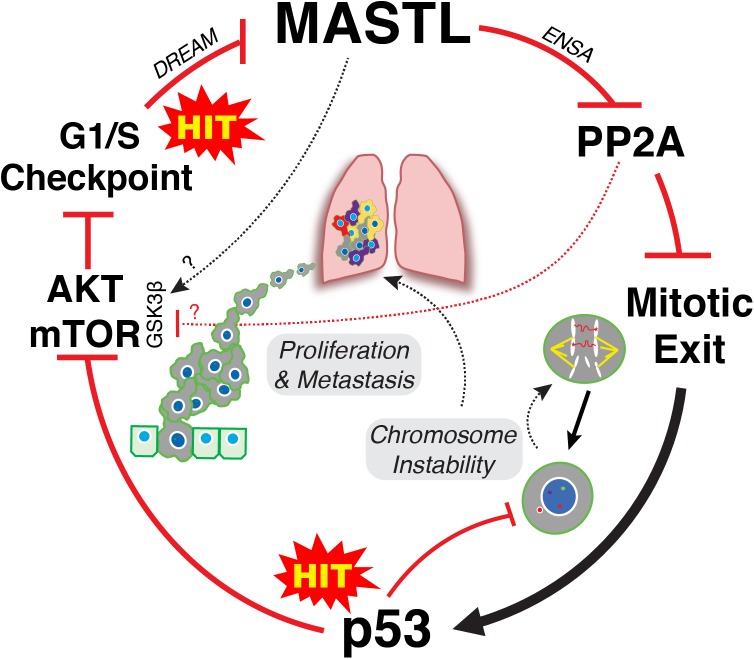
Two-hit model of MASTL-driven cancer evolution. Hit one occurs at the G1/S level leading to disruption of DREAM and potentially E2F8 mediated increased transcription of MASTL. Overexpression of MASTL then maintains phosphorylation of ENSA, delaying the correct timing of PP2A-B55 reactivation during mitotic exit leading to increased mis-segregation of chromosomes. A second hit that mutates p53 then allows these defective cells to continue to proliferate, thereby creating further mitotic defects and ongoing CIN, driving tumor heterogeneity. In parallel, overexpression of MASTL deregulates AKT/mTOR signaling, driving proliferation and metastasis. The mechanism for deregulation of AKT/mTOR signaling are still unclear but appears to involve increased phosphorylation of S9 on GSK3β. This could be through inhibition of PP2A-B55, or may involve MASTL phosphorylation of novel, unknown substrates.

It is clear that upregulation of MASTL and or loss of PP2A-B55 can promote CIN, cancer growth and invasion. However, an explanation for how MASTL in particular is upregulated by cells remains a mystery. A recent publication by Pfister et al proposed an inviting model for how overexpression of mitotic genes can drive CIN in breast cancer (Pfister et al., 2018). Specifically, simply overexpressing the mitotic gene transcription factors E2F1, MYBL2, FOXM1, and DREAM (DP, RB-like, E2F4, and MuvB), significantly increased the rate of mitotic defects and micronuclei in *Xenopus* embryos. This provides an elegant explanation for how mitotic gene overexpression drives CIN without the need for direct mutation. While the MASTL transcription factor is currently unknown, MASTL is a potential DREAM target (Fischer et al., 2016) and recent evidence also suggests that MASTL transcription can be increased by E2F8 in ER positive breast cancer cells (Tian et al., 2017). E2F8 was noted by Pfister et al to strongly correlate with high levels of functional aneuploidy in tumor samples (Pfister et al., 2018). In addition, they also noted that overexpression of mitotic genes strongly correlated with p53 mutations in breast cancer, a correlation we also observed with MASTL overexpression (Rogers et al., 2018; Figure 1B). Normally, p53 would arrest defective mitotic cells in the following G1 phase, thereby preventing further proliferation (Hinchcliffe et al., 2016). Consequently, mutating p53 provides a significant growth advantage by allowing defective cells to continue proliferating. This in turn drives further mitotic errors resulting in ongoing CIN and increased tumor heterogeneity (Figure 2).

Taken together, we propose a classic two hit model for how MASTL overexpression drives cancer. One hit occurs at the level of cell cycle gene transcription resulting in MASTL overexpression. Disruption of gene transcription is a hallmark of cancer that can occur through multiple mechanisms, such as oncogenic growth factor signaling, oncogenic viruses, loss of tumor suppressors like Rb, p16 and PTEN or as shown above, through increased expression of oncogenic transcription factors (Hanahan and Weinberg, 2011). Increased MASTL expression then disrupts timely reactivation of PP2A-B55 during mitotic exit (Rogers et al., 2018), leading to mis-segregation of chromosomes and aneuploidy (Figure 2). A second hit to p53 allows these aneuploid cells to continue proliferating, which in turn enables and promotes further CIN. In parallel, MASTL overexpression disrupts AKT/mTOR and potentially Wnt/β-catenin signaling, promoting invasion and metastasis (Figure 2). In combination, this produces tumors that are highly proliferative, unstable and malignant resulting in reduced patient survival.

## Targeting Mastl in Cancer

Inhibition of MASTL as a therapeutic strategy for cancer has received growing interest in the last few years, with potential for both single agent therapy and combination with current standard of care treatments. As a single agent, MASTL inhibitors could be used to reduce proliferation and metastasis in cancers, such as triple negative breast cancer (TNBC), where MASTL is significantly overexpressed. In support, knockdown of MASTL in the TNBC MDA-MB-231 breast cancer cell line, blocked tumor growth and metastasis *in vivo* (Rogers et al., 2018). Similarly, CRISPR knockout or RNAi knockdown of MASTL also reduced growth of some but not all breast cancer cell lines *in vitro* and *in vivo* (Vera et al., 2015; Álvarez-Fernández et al., 2018), suggesting that additional biomarkers will be needed for successful monotherapy. Importantly, knockdown of MASTL reduced viability of thyroid cancer cells without significantly affecting normal cell proliferation (Anania et al., 2015), suggesting that MASTL inhibitors may be relatively non-toxic. The lack of toxicity is notable given that MASTL knockout mice are embryonically lethal (Álvarez-Fernández et al., 2013), and a point mutation of MASTL has been linked with thrombocytopenia (Gandhi et al., 2003; Johnson et al., 2009; Hurtado et al., 2018). Taken together, this suggests that although MASTL inhibition possesses the potential for significant side-effects in patients, there might be a therapeutic window for inhibition of MASTL with small molecules. In support, the mitotic state can be maintained in Xenopus extracts that have up to ∼80% of MASTL depleted (Vigneron et al., 2011), indicating that a small fraction of active MASTL might be sufficient for normal proliferating cell homeostasis.

The second possibility for targeting MASTL is in combination with DNA damaging agents. The rationale for this is based on results showing that MASTL is critical for promoting checkpoint recovery from DNA damage (Peng et al., 2010, 2011; Wong et al., 2016). Consequently, overexpression of MASTL has been associated with resistance to cisplatin (Wang et al., 2014) by accelerating checkpoint recovery (Wong et al., 2016). Conversely, knockdown of MASTL can sensitize cancer cells to cisplatin, radiotherapy and 5-fluorouracil (5FU) in several cancer types (Wang et al., 2014; Nagel et al., 2015; Uppada et al., 2018; Yoon et al., 2018), most likely by preventing cells from re-starting the cell cycle following damage. However, it is likely that combination of MASTL inhibitors with mitotic chemotherapies will have the opposite effect. MASTL knockdown was shown to be strongly antagonistic with paclitaxel (Swanton et al., 2007) as it promotes mitotic slippage and polyploidy. Interestingly, overexpression of mitotic genes along with high levels of CIN are linked with resistance to paclitaxel in breast cancer (Swanton et al., 2009), suggesting that overexpressed MASTL could potentially be used as a biomarker for resistance to paclitaxel and other mitotic chemotherapies.

In summary, it is clear that under specific conditions MASTL is a highly promising target for several cancers including breast, lung, colon and ovarian. With the recent identification of a first-generation MASTL inhibitor (Ocasio et al., 2016), the potential for a future therapeutic breakthrough is looking promising and exciting.

## Author Contributions

AB conceived, co-wrote the manuscript and produced the artwork for Figure 2. KM co-wrote the manuscript.

## Conflict of Interest Statement

The authors declare that the research was conducted in the absence of any commercial or financial relationships that could be construed as a potential conflict of interest.
